# Special Issue “Recent Developments in Bio-Based Particleboards and Fiberboards”

**DOI:** 10.3390/ma18245556

**Published:** 2025-12-11

**Authors:** Philippe Evon

**Affiliations:** Laboratory of Agro-Industrial Chemistry (LCA), French National Research Institute for Agriculture, Food and Environment (INRAE), Toulouse INP, Université de Toulouse, 31030 Toulouse, France; philippe.evon@toulouse-inp.fr

## 1. Introduction and Scope

This Special Issue, “Recent Developments in Bio-Based Particleboards and Fiberboards”, provides an inventory of the latest research in the area of composites reinforced with natural fibers and more particularly (but not exclusively) particleboards and fiberboards.

Plant fibers have many advantages. They are abundant and cheap, and they have a reduced impact on the environment while not competing with the food industry for land use [1]. Whether thermoplastics or thermosets, binders can also be of biosourced origin, namely, lignins [2], starch [3], or proteins [4]. Bio-based particleboards and fiberboards can thus be independent from fossil resources, with the advantage of being low VOC emitters [5]. In particular, the replacement of formaldehyde-based resins with natural binders makes them less harmful to the environment and human health.

The topics covered in the Special Issue include the origin of natural fibers and binders, fiber preparation, mixture preparation and molding, water-proofing strategies to improve the material’s water resistance, thermo-mechanical performance (including in humid environments), and associated uses.

## 2. An Overview of the Published Articles

This Special Issue includes five research reports by authors from four European countries, namely, France, Germany, Poland, and Spain.

For the first article [6], *Sargassum* spp. algae were used to develop self-bonding panels using thermocompression. These algae have been proliferating in the Atlantic Ocean since 2010, stranding on Caribbean beaches, and causing significant economic, environmental, and health problems. In their research, Bauta et al. developed a high-density, binder-free particleboard using an innovative process combining twin-screw extrusion for the preparation of *Sargassum* algae simultaneously with the removal of sea salt and uniaxial thermocompression coupled with an original cooling system for shaping. The influence of the experimental thermocompression conditions (i.e., temperature, pressure, and duration) on the performance of the particleboards was studied. Under optimal thermocompression conditions (i.e., 200 °C, 40 MPa, and 7.5 min), a high-density panel (1.63 g/cm^3^) was produced, exhibiting particularly high flexural strength, namely, a stress at break of 32.3 MPa and an elastic modulus of 6.8 GPa. Its contact angle with water was, however, rather low (38.9°), limiting its subsequent use exclusively to dry environments. As evidenced by thermal analyses, the alginates from *Sargassum* algae contributed to self-bonding. This work paves the way for an original application of *Sargassum* algae in the field of boards (furniture, packaging, construction, etc.), using the whole algae with minimal pre-treatment, without the need to add any synthetic resin before shaping.

For the second article, by Cavailles et al., sugarcane bagasse (SCB), an agro-industrial by-product widely available on Reunion Island in France, was used to produce self-bonding fiberboards, again using thermocompression [7]. The influence of five thermocompression conditions (i.e., pressure, temperature, duration, fiber-to-fine-particle ratio, and moisture content) was evaluated, and these parameters influenced the properties of the fibrous materials obtained with a density ranging from 1198 to 1507 kg/m^3^, a flexural strength ranging from 6.1 to 43.6 MPa, a flexural modulus ranging from 0.9 to 6.9 GPa, and a thickness swelling after 24 h immersion in water ranging from 9% to 208%. The presence of fine particles in SCB combined with a low moisture content (4–10%) and high temperatures (≥200 °C) and pressures (≥68 MPa) increased the mechanical properties, accompanied by an increase in density. At the same time, under more severe conditions (i.e., a temperature of 240 °C, a duration of 30 min, and humidity ≥ 12.5%), water resistance was improved, due to lignin plasticization on the one hand, and internal chemical reorganization followed by hydrolysis of hemicelluloses into water-soluble extracts on the other. The boards obtained could be used in the furniture or construction industries, particularly as P2-type panels, corresponding to panels used in dry environments for interior fittings and furniture, in accordance with standard NF EN 312 [8].

For the third article, Moll et al. used two other agricultural sources, i.e., *Miscanthus* and *Paulownia*, which are perennial crops, for the hot-pressing manufacturing of self-bonding panels [9]. As in the two previous research reports [6,7], these biomasses are more sustainable alternatives to limited wood resources, such as *Picea*, which was also studied as a reference in [9]. During their work, the authors highlighted the influence of particle size and of the mixture of particles of different sizes on the mechanical strength of the boards produced. These boards exhibited normal ignitability, as defined by Euroclass E according to standard EN13501-1 [10]. With higher proportions of finer particles in the mixtures, the materials obtained were denser and also exhibited improved mechanical performance. Panels made from the finest *Miscanthus* mixtures and all those based on *Paulownia* met the requirements of standard EN 622-2 [11] in terms of elastic modulus, making their use feasible in load-bearing dry applications in the construction sector.

In the fourth article, Frydrysiak demonstrate, through an upcycling approach, the possibility of using keratin waste to produce high-value-added products [12]. This has resulted in the creation of multi-layer textile laminates based on keratin flour. These laminates offered good thermal insulation properties (i.e., 0.038–0.040 W/mK for thermal conductivity) in comparison with conventional materials, as well as consistent comfort for use in construction. In addition, Frydrysiak’s work paves the way for the recovery of keratin powder, a waste product from the poultry processing industry originating from the grinding of chicken feathers. Poorly valued, the management of this waste is often problematic due to the large quantities that need to be treated and the release of pollutants into the environment (e.g., the spread of odors and pathogens in soil and water). Its use in manufacturing thermal insulation materials for the construction sector supports the transition to a circular economy.

Last but not least, barley straw, an agricultural by-product largely available in Spain, was used in the fifth and final article as a source of cellulosic fibers to mechanically reinforce polyhydroxy-3-butyrate (PHB) inside PHB-based biocomposites [13]. In this work from Oliver-Ortega et al., barley straw was first pre-treated via mechanical defibration to accurately separate the fibers (DFBF) via delignification using soda (100 °C for 2.5 h with NaOH at 7% (*w*/*w*) in proportion to fibers) (DBF) and even via the subsequent bleaching of DBF with hydrogen peroxide (70 °C for 3 h with H_2_O_2_ at 20% (*w*/*w*) in proportion to fibers) (BBF). These three fillers were studied for their ability to mechanically reinforce PHB and were compared to commercial wood flour. Despite the absence of matrix-to-fiber interface coupling, the DFBF-containing composites exhibited mechanical properties similar to those of neat PHB. The mechanical defibrating of barley straw to produce DFBF yielded individual fibers without the addition of chemicals, did not alter the chemical composition of barley straw, and was considered the most eco-friendly method of producing the filler. With a view to intensifying the mixing process (first performed in batch in an internal mixer), pellets combining PHB with DFBF, added up to 30% (*w*/*w*), were produced in a continuous mode by compounding in a twin-screw extruder and subsequently injected. Their automatic injection molding demonstrated the ability to easily produce parts for agriculture applications, such as flowerpots, on a large scale, and finite analysis simulation (FEA) confirmed the suitability of the PHB/DFBF (70/30) biocomposite for such an application.

## 3. Conclusions

In this Special Issue, titled “Recent Developments in Bio-Based Particleboards and Fiberboards”, including five research reports, we provide an overview of the latest research in the field of natural fiber-reinforced composites. These composites can be binderless particleboards or fiberboards, biolaminates based on keratin flour, a significant by-product of the poultry processing and meat industries, or PHB-based composite materials reinforced with cellulosic fibers and designed to be molded via plastic injection into parts for agriculture applications.

This Special Issue will be of particular interest to producers of bio-based composite materials, especially those intended for the furniture, packaging, and construction sectors (e.g., fiberboards, and particleboards), with the aim of bringing more environmentally friendly materials onto the market in the future.
